# Changes of intracardiac flow dynamics measured by HyperDoppler in patients with aortic stenosis

**DOI:** 10.1093/ehjopen/oeae069

**Published:** 2024-08-08

**Authors:** Jolanda Sabatino, Isabella Leo, Antonio Strangio, Sabrina La Bella, Rosalba De Sarro, Vincenzo Montemurro, Gianni Pedrizzetti, Fabio Troilo, Marco Maglione, Daniele Torella, Giovanni Di Salvo, Salvatore De Rosa

**Affiliations:** Department of Experimental and Clinical Medicine, Magna Graecia University, Catanzaro, Italy; Pediatric Research Institute (IRP) ‘Città della Speranza’, Padua, Italy; AOU Renato Dulbecco, Viale Europa, 88100 Catanzaro, Italy; Department of Women’s and Children’s Health, University Hospital Padua, Padua, Italy; Department of Experimental and Clinical Medicine, Magna Graecia University, Catanzaro, Italy; AOU Renato Dulbecco, Viale Europa, 88100 Catanzaro, Italy; Department of Experimental and Clinical Medicine, Magna Graecia University, Catanzaro, Italy; AOU Renato Dulbecco, Viale Europa, 88100 Catanzaro, Italy; AOU Renato Dulbecco, Viale Europa, 88100 Catanzaro, Italy; Department of Experimental and Clinical Medicine, Magna Graecia University, Catanzaro, Italy; Department of Medical and Surgical Sciences, Magna Graecia University, Viale Europa, 88100 Catanzaro, Italy; Department of Engineering and Architecture, University of Trieste, Trieste, Italy; GLM Esaote Spa, 16155 Genova, Italy; GLM Esaote Spa, 16155 Genova, Italy; Department of Experimental and Clinical Medicine, Magna Graecia University, Catanzaro, Italy; AOU Renato Dulbecco, Viale Europa, 88100 Catanzaro, Italy; Pediatric Research Institute (IRP) ‘Città della Speranza’, Padua, Italy; Department of Women’s and Children’s Health, University Hospital Padua, Padua, Italy; AOU Renato Dulbecco, Viale Europa, 88100 Catanzaro, Italy; Department of Medical and Surgical Sciences, Magna Graecia University, Viale Europa, 88100 Catanzaro, Italy

**Keywords:** Aortic stenosis, Fluid dynamics, Vortices

## Abstract

**Aims:**

Assessment of intracardiac flow dynamics has recently acquired significance due to the development of new measurement methods based on echocardiography. Recent studies have demonstrated that cardiac abnormalities are associated with changes in intracardiac vortical flows. Yet, no previous study assessed the impact of aortic stenosis (AS) on intracardiac vortices. This study aims to explore the clinical potential of additional information provided by quantifying intracardiac flow dynamics in patients with AS.

**Methods and results:**

One hundred and twenty patients with severe AS, sixty patients with concentric ventricular remodelling (VR), and hundred controls (CTRL) were prospectively included and underwent non-invasive evaluation of intracardiac flow dynamics. In addition to standard echocardiography, fluid dynamics were assessed by means of HyperDoppler. Vortex depth (*P* < 0.001), vortex length (*P* = 0.003), vortex intensity (*P* < 0.001), and vortex area (*P* = 0.049) were significantly increased in AS compared with CTRL. In addition, mean energy dissipation was significantly higher in AS compared with CTRL (*P* < 0.001) and VR (*P* = 0.002). At receiver operating characteristic analysis, vortex depth showed the best discrimination capacity for AS (*P* < 0.001).

**Conclusion:**

Changes in fluid dynamics–based HyperDoppler indices can be reliably assessed in patients with AS. Significant changes in vortex depth and intensity can selectively differentiate AS from both concentric remodelling and healthy CTRLs, suggesting that the assessment of intracardiac flow dynamics may provide complementary information to standard echocardiography to better characterize patients’ subsets.

## Background

Intracardiac fluid dynamics were first described by Leonardo Da Vinci, as documented in his famous drawings. Recent technical developments ignited novel interest, thanks to the ability to portraying of vortices inside cardiac chambers and particularly in the left ventricle (LV),^1,2^ even though their application is rapidly wide spreading to other chambers/valves.^3^

A vortex is a fluid phenomenon characterized by a typical swirling motion around an imaginary central axis.^4^ Among their interesting features is the ability to behave as a reservoir of kinetic energy (KE). During early diastole, the trans-mitral flow creates two main vortices originating from the free margins of mitral valve (MV) leaflets. The geometrical imbalance of MV leaflets favours the generation of a main anterior vortex rotating clockwise and a smaller posterior vortex rotating counterclockwise.^3,4^ During atrial systole, an additional vortex takes shape that redirects the blood streams towards LV outflow tract (LVOT).

Over the past few years, advances in cardiac magnetic resonance imaging (MRI) and contrast echocardiography allowed to visualize and to quantify intracardiac vortices, promising results for some clinical applications,^5–8^ including heart failure,^5,6^ cardiac valve dysfunction,^7^ and congenital heart disease.^8^ However, these technologies have not yet entered clinical routine, being time-consuming, with limited availability and higher costs and requiring specific expertise. Lately, echocardiographic techniques based on semi-automated analysis and not requiring contrast agents were introduced, which are able to measure intracardiac fluid dynamics.^3^

Aortic stenosis (AS) is the most prevalent valvular heart disease in Western countries especially in older adults. If left untreated, the appearance of the first symptoms marks a high mortality risk.^9^ Assessing AS severity by two-dimensional echocardiography is challenging and might be misclassified in a non-negligible proportion of patients.^10,11^ Interestingly, aortic valve stenosis may affect the formation of the trans-mitral vortex in a unique form, by means of the flow restriction across the valve.

Therefore, the aim of this study was to characterize the intracardiac flow dynamics of patients with severe AS compared with patients with concentric remodelling and healthy volunteers, by using the non-contrast echocardiographic technique based on colour Doppler flow mapping (CDFM). Understanding the impact of AS on intracardiac vortices will provide essential knowledge to explore potential clinical applications of vortex-based indices in these patients.

## Methods

### Study population

Consecutive patients with AS were prospectively enrolled at the Magna Graecia University of Catanzaro. Inclusion criteria are as follows: age > 18 years old; established diagnosis of high-gradient AS [mean gradient ≥ 40 mmHg, peak velocity ≥ 4.0 m/s, valve area ≤ 1 cm^2^ (or ≤0.6 cm²/m²)] or low-flow low-gradient (LFLG) AS (mean gradient < 40 mmHg, valve area ≤1 cm^2^, Stroke Volume index ≤ 35 mL/m^2^). Low-dose dobutamine stress echocardiography (DSE) was performed in the latter subset of patient to identify true severe AS and exclude the presence of pseudo-severe AS. Exclusion criteria are as follows: concomitant haemodynamically significant valvular heart disease; valvular prosthesis; ongoing arrhythmias, including atrial fibrillation, or ventricular dyssynchrony due to bundle branch block or paced rhythm; and poor acoustic window. A parallel subset of patients with no significant structural heart disease but with LV concentric ventricular remodelling (VR) were consecutively included to differentiate the fluid dynamics impact of AS itself from the accompanying LV remodelling. Ventricular remodelling was defined as absence of LV hypertrophy (LVH), as defined by the LV mass index (95 g/mq for women and 115 g/mq for men), and a relative wall thickness (RWT) ratio > 0.42, while normal geometry is defined as no LVH with a RWT ratio ≤ 0.42. Finally, a parallel group of control subjects without AS (CTRL) was also included. Qualification for CTRL included the absence of any significant structural nor functional/structural LV alteration. All patients gave informed consent. The study protocol was approved by the Ethics Review Board. Each patient underwent a complete echocardiographic examination at baseline. Patient demographics and clinical and laboratory data were collected for each patient.

### Echocardiographic assessment

Comprehensive transthoracic echocardiography was performed using Esaote MyLab™ X8 Platform (Esaote S.P.A, Genova, Italy) using a 1–5 MHz electronic phased array transducer. Left ventricular end-diastolic diameter, LV end-systolic diameter, interventricular septum, LV mass, LVOT diameter, pulmonary artery systolic pressure, LV diastolic parameters [peak early (E) and late (A) diastolic velocities on trans-mitral flow, septal and lateral e, and average E/e′ ratio], left atrial volume index, LV function by Simpson biplane, and right ventricular S′ were measured for each patient. Peak transaortic pressure gradient and mean pressure gradient across the aortic valve were measured by continuous wave Doppler. The velocity–time integral and stroke volume were instead calculated on the pulsed wave Doppler recordings of the LVOT. Aortic valve area (AVA) was derived using the continuity equation, with an AVA ≤ 1 cm^2^ or indexed AVA < 0.6 cm^2^/m^2^ identifying severe AS, as previously described.^10^

### Fluid dynamics analysis

**Figure 1:**
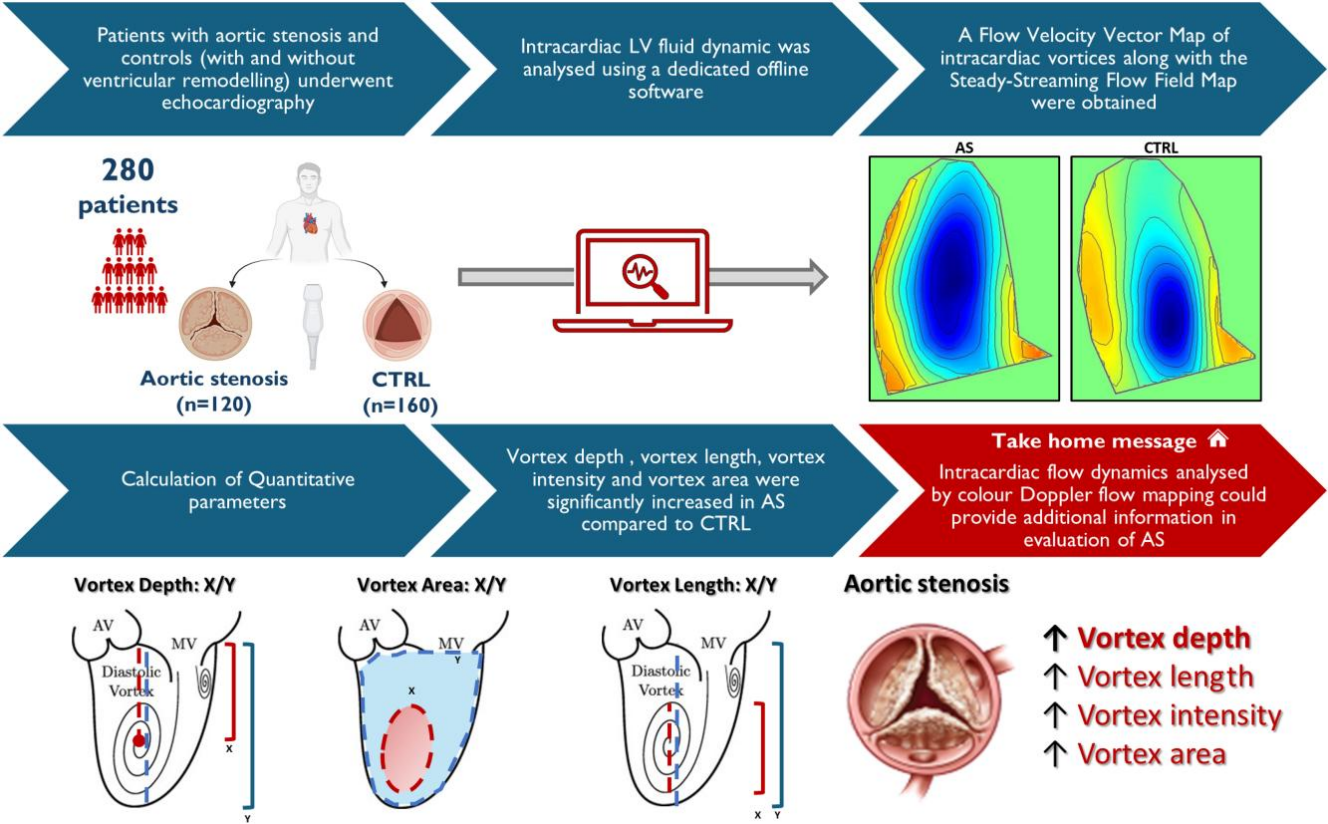
Depiction of the two main study groups (upper part) with representative images depicting the geometrical appearance of intracardiac vortex lines in patients with aortic stenosis or controls (upper right). The lower part of the figure summarizes the main study findings. Some of the graphical elements were created with BioRender.com (2023).

For intracardiac LV fluid dynamics analysis, a three-chamber apical view was acquired at hold end-expiration in a cine loop format, including two consecutive cardiac cycles, with a frame rate between 23 and 25 Hz and analysed by a CDFM software analysis (HyperDoppler v. 1.0.3, Esaote, Genova, Italy). Particular attention was taken to include most of the LV cavity and the LVOT within the colour Doppler sector angle and with a pulse repetition frequency of 4.4 MHz. The acquired images were subsequently exported as DICOM files for storage and further analyses (HyperDoppler v. 1.0.3, Esaote, Genova, Italy). The endocardial border was manually contoured at end-diastole and automatically tracked during the whole cardiac cycle. Thus, intracardiac flow dynamics are registered during the entire cardiac cycle, allowing the construction of steady-streaming flow of one entire cycle, depicting streamlines and colour-coded maps of intracardiac vortices, as illustrated in the *Figure 1*. Briefly, LV vortices start forming at the beginning of diastole, as blood enters the LV from the atrium. The main anterior vortex rotates clockwise and the secondary posterior vortex counterclockwise. As vortices move towards the LV apex, they deform due to pressure gradients and interactions with LV walls. Finally, during isovolumic contraction, the vortex redirects blood towards the LVOT, forming a large anterior vortex, and blood is ejected. Rather than describing the vortex properties at specific instants during the heartbeat, the HyperDoppler software reproduces the steady-streaming flow field (reflecting the heartbeat-averaged flow) and evaluates the properties of the steady-streaming vortex. This approach provides a global measure of the vortex pertaining to the entire heartbeat.

In addition, the following quantitative parameters were calculated^3^:

- *Localization parameters.* Vortex depth (VD): the distance of the vortex centre from the LV base divided by total LV long axis; vortex length (VL): the ratio between the vortex longitudinal length and the total LV length; vortex width: the ratio between the horizontal width of the vortex and the total LV width; vortex area (VA): the ratio between the VA and the total LV area.
- *Parameters of vorticity.* Vortex intensity (VI): the sum between the clockwise and counterclockwise vortex circulation. The latter is calculated as the integral of vorticity inside the vortex.
- *Energy parameters.* Kinetic energy dissipation (KED): the amount of KE dissipated in the LV during the whole cardiac cycle. This represents an indirect efficiency parameter; in fact, the lower the DKE is, the more efficient the system is; KE fluctuation (KEF): the ratio between the standard deviation of the KE and the average KE; vorticity fluctuation index: the ratio between the variance of vorticity and the average square vorticity.
- *Pressure gradient parameters.* Flow force angle (FFA): the dominant direction of flow momentum (normally between 0 for longitudinal flow momentum and 90 for transversal flow momentum).
- *Flow transit parameters.* Direct flow (DF): the amount of the LV end-diastolic volume that during one cardiac cycle goes directly from inlet to outlet.

All echocardiographic analyses were blindly and independently performed by two operators.

### Statistical analysis

Statistical analyses were performed using SPSS version 25.0 (IBM, Armonk, NY, USA). Continuous variables are expressed as mean ± SD and as median (interquartile range), according to the distribution of the variable. Categorical variables are reported as frequency and percentage. Reproducibility of measurements was assessed on a randomly selected subgroup of 54 exams. Two operators independently performed flow analysis on different acquisitions. Intra- and inter-observer variability is expressed as the mean percentage error (absolute difference/mean), as well as using the intraclass correlation coefficient. Differences between groups were assessed using the unpaired Student’s *t*-test, the Mann–Whitney U-test, and the Pearson *χ*^2^ test, as appropriate, considering *P* < 0.05 as statistically significant. The ability of the fluid dynamics parameters to distinguish patients with severe AS was assessed using the receiver operating characteristic (ROC) curve examining the area under the curve (AUC) with 95% confidence interval.^12^ The optimal threshold value (cut-off point) was determined using the maximization of the Youden index method.

## Results

### Baseline characteristics

We included 280 patients, specifically 120 patients (65 females, 54%) with severe AS, 100 (32 females, 32%) CTRLs, and 60 patients (7 females, 12%) with LV concentric remodelling in absence of other cardiac anomalies (VR). Demographic and clinical characteristics of the study population are shown in *Table 1*. Hundred patients (83.3%) of the AS cohort had concomitant arterial hypertension, 41.7% diabetes mellitus, and 24.2% coronary artery disease. All patients were on optimal medical treatment based on practice guidelines. Baseline population characteristics are summarized in *Table 1*.

**Table 1:** Study population

	CTRL	AS	VR
Age (years)	54.5 ± 16.2	80.2 ± 7.0^a^	63.0 ± 10.5^a,b^
Female sex	32 (32%)	65 (54.2%)	7 (11.7%)
BSA (m^2^)	1.86 ± 0.19	1.77 ± 0.17^a^	1.88 ± 0.16^b^
Hypertension	47 (47%)	100 (83.3%)^a^	49 (81.7%)
Smoke	14 (14%)	11 (9.2%)	12 (20%)
Diabetes mellitus	15 (15%)	50 (41.7%)^a^	20 (33.3%)
Dyslipidaemia	50 (50%)	83 (69.2%)	46 (76.6%)
CAD	34 (34%)	29 (24.2%)	35 (58.3%)
Stroke	2 (2%)	9 (7.5%)	0
Creatinine (mg/dL)	0.96 ± 0.59	1.15 ± 0.72^a^	1.17 ± 1.38^b^
Hb (g/dL)	13.96 ± 1.59	12.09 ± 1.65^a^	13.79 ± 1.85^b^
PLT (×10^3^/µL)	209.39 ± 54.44	210.99 ± 73.31	212.11 ± 78
NT-proBNP (pg/mL)	97 ± 87.95	2286.8 ± 3090.67^a^	107 ± 174.5^b^

CTRL, controls; AS, patients with severe aortic stenosis; VR, subjects with left ventricular remodelling; BSA, body surface area; CAD, coronary artery disease (history); Hb, haemoglobin; PLT, blood platelet count; NT-proBNP, *n*-terminal pro-brain natriuretic peptide.

^a^ *P* < 0.05 compared with CTRL.

^b^ *P* < 0.05 compared with AS.

### Standard echo parameters

In the AS group, mean AVA was 0.71 ± 0.18 cm², and the transaortic gradient was 50.63 ± 14.73 (*Table 2*). Patients from the AS group showed a slightly reduced LV ejection fraction (LVEF) compared with the VR group (*P* < 0.001) or CTRL (*P* < 0.001). Right ventricular S′ was significantly reduced in AS compared with CTRL (*P* = 0.001). E wave/A wave (E/A) was reduced (*P* = 0.019) and the mean E/E′ ratio increased (*P* < 0.001) in AS compared with CTRL. A more detailed description of echocardiographic parameters is reported in *Table 2*.

**Table 2:** Echocardiographic characterization

	CTRL	AS	VR
LV mass indexed	83.7 ± 16.1	130.0 ± 24.0^a^	91.7 ± 16.6^a,b^
RWT	0.34 ± 0.08	0.46 ± 0.12^a^	0.46 ± 0.05^a^
EF (%)	58.0 ± 4.0	52.6 ± 7.7^a^	56.7 ± 4.8^b^
LAVi (mL/m^2^)	28.0 ± 7.1	45.2 ± 13.8^a^	31.6 ± 7.7^a,b^
LV EDD (mm)	49.0 ± 4.2	49.8 ± 5.5	46.1 ± 4.0^a,b^
LV ESD (mm)	34.1 ± 5.0	34.7 ± 5.3	32.6 ± 5.3
LV PWT (mm)	8.9 ± 1.1	11.3 ± 1.2^a^	10. 6 ± 0.9^a,b^
IVST (mm)	9.4 ± 1.2	12.5 ± 1.5^a^	11.2 ± 1.0^a,b^
RV S′ (cm/s)	13.2 ± 1.8	11.8 ± 2.7^a^	12.5 ± 1.9
sPAP (mmHg)	27.3 ± 4.4	38.8 ± 9.5^a^	29.3 ± 5.1^a,b^
E/A	1.03 ± 0.33	0.89 ± 0.39^a^	0.92 ± 0.24^a^
E/e′ mean	7.27 ± 2.24	12.9 ± 3.55^a^	8.34 ± 2.14^a,b^
Mean gradient (mmHg)	3.89 ± 1.77	50.63 ± 14.73^a^	4.12 ± 1.15^b^

CTRL, controls; AS, patients with severe aortic stenosis; VR, subjects with left ventricular remodelling; LV, left ventricle; RWT, relative wall thickness; EF, ejection fraction; LAVi, left atrial volume index; EDD, end-diastolic diameter; ESD, end-systolic diameter; PWT, posterior wall thickness; IVST, interventricular septum thickness; RV S′, right ventricle S′ wave; sPAP, estimated systolic pulmonary arterial pressure; E/A, mitral E wave to A wave ratio; E/e′ mean, mitral E wave to e′ wave.

^a^ *P* < 0.05 compared with CTRL.

^b^ *P* < 0.05 compared with AS.

### Vortex localization and parameters of vorticity

**Figure 2:**
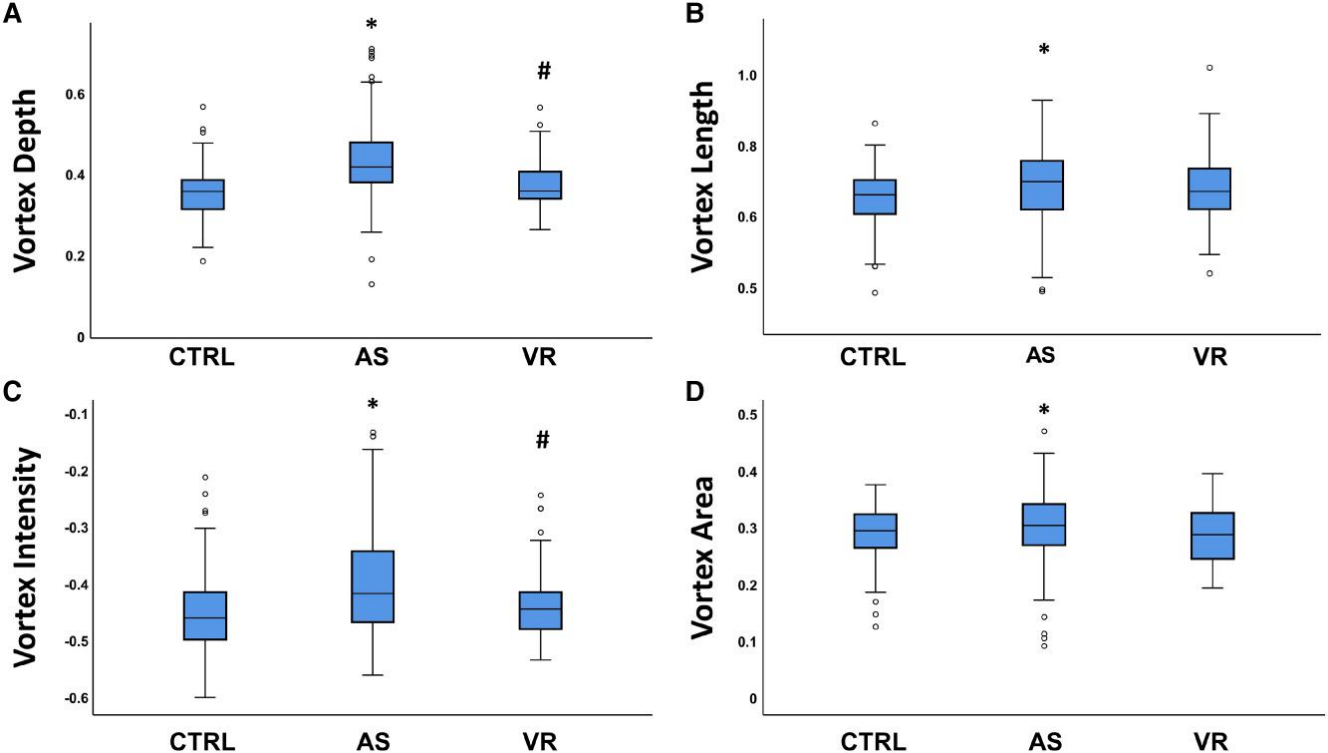
Geometrical and vorticity parameters. Measured value of vortex depth (*A*), vortex length (*B*), vortex intensity (*C*), and vortex area (*D*) in all study groups: controls, patients with severe aortic stenosis, and patients with left ventricular concentric remodelling. *P  *<* 0.05 compared with control; ^#^*P*  *<* 0.05 compared with aortic stenosis.

Vortex depth (*P* < 0.001), VL (*P* = 0.003), VI (*P* < 0.001), and VA (*P* = 0.049) were significantly increased in AS compared with CTRL (*Figure 2*). In line with this finding, VD (*P* < 0.001) and VI (*P* = 0.013) were significantly higher in AS compared with VR patients.

### Energy and pressure gradient parameters

**Figure 3:**
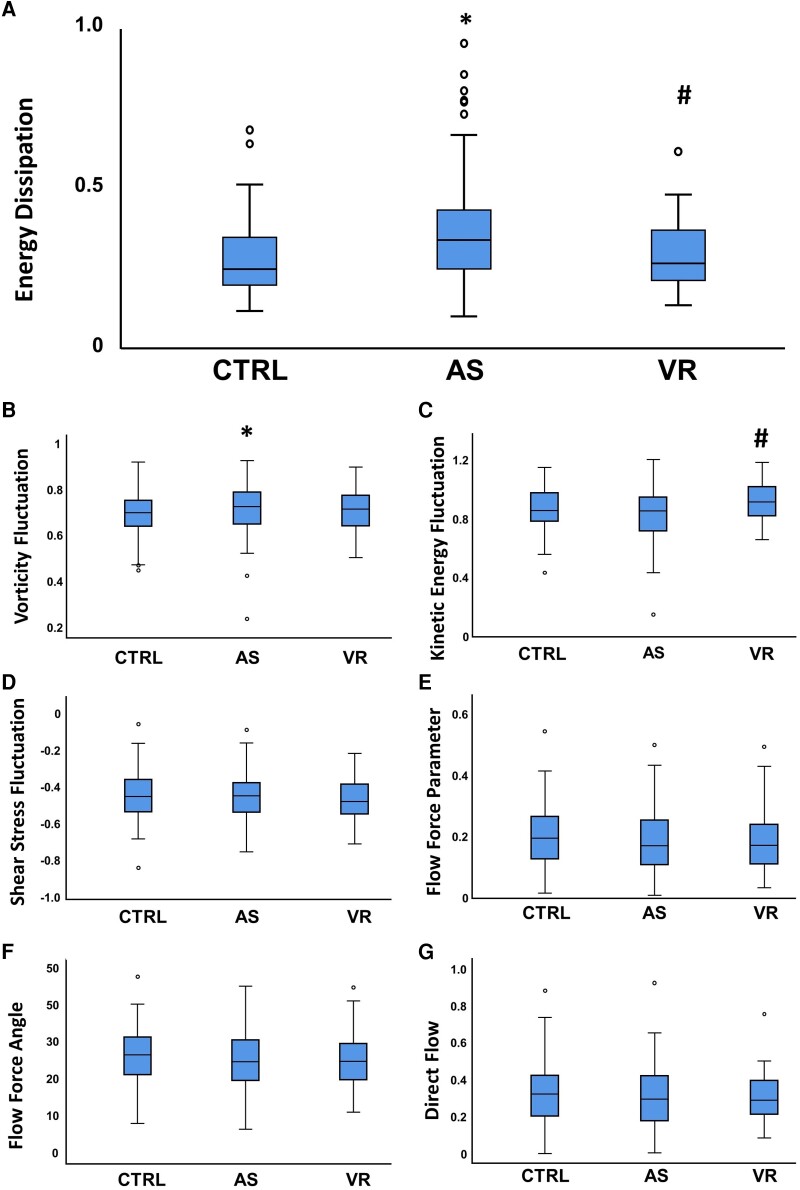
Energy, pressure, and flow transit parameters. Measured value of energy dissipation (*A*), vorticity fluctuation (*B*), kinetic energy fluctuation (*C*), shear stress fluctuation (*D*), flow force parameter (*E*), flow force angle (*F*), and direct flow (*G*) in all study groups: controls, patients with severe aortic stenosis, patients with left ventricular concentric remodelling. *P  *<* 0.05 compared with control; ^#^*P*  *<* 0.05 compared with aortic stenosis.

The mean energy dissipation of AS group was significantly increased compared with the CTRL (*P* < 0.001) (*Figure 3*) and VR group (*P* = 0.002). Moreover, vorticity fluctuation was significantly increased in AS compared with CTRL (*P* = 0.028), with no difference compared with VR (*P* = 0.486) Furthermore, KEF was significantly increased in VR compared with AS (*P* = 0.004), but no difference was found in AS compared with CTRL. No significant variations in terms of shear stress fluctuation, flow force parameter, and FFA were observed.

### Flow transit parameters

No significant difference in DF was found in AS compared with CTRL (*P* = 0.470) or VR (*P* = 0.974).

### Receiver operating characteristic curve

**Figure 4:**
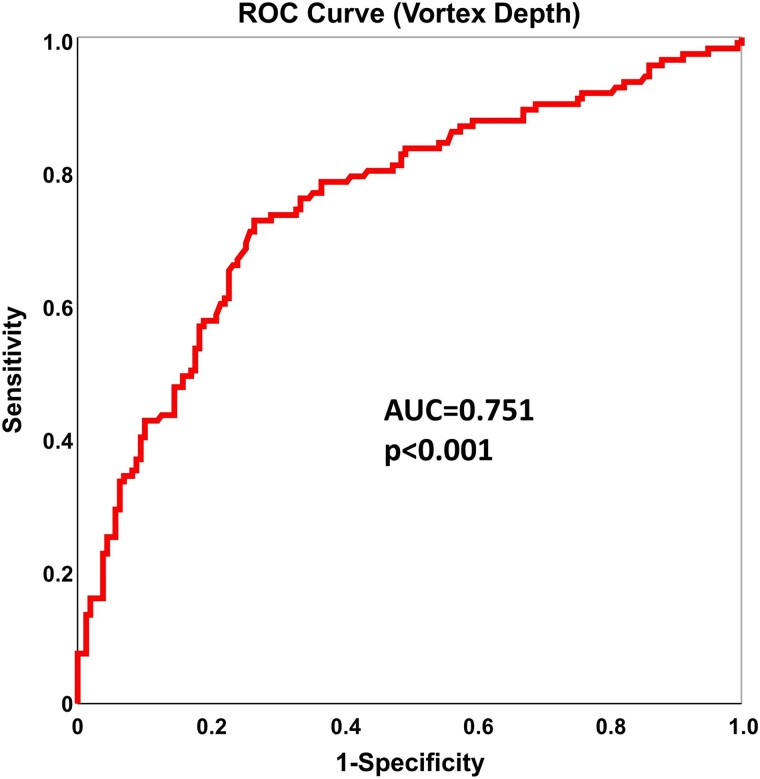
Diagnostic performance of vortex depth. Receiver operating characteristic analysis depicting the diagnostic performance of vortex depth to discriminate patients with severe aortic valve stenosis.

Among the fluid dynamics parameters, VD showed the best diagnostic performance (AUC of 0.751; *P* < 0.001) to discriminate patients with severe AS at ROC curve analysis (cut-off value ≥ 0.354; sensitivity, 73%; specificity, 73%) (*Figure 4*).

### Reproducibility analyses

Intra- and inter-observer variability was excellent for VA, VD, VL, and energy dissipation; very good for VI, vorticity fluctuation, shear stress fluctuation, flow force parameter, and FFA; and poor for DF measurement, as reported in more details in Supplementary material online, *Table S1*. Bland–Altman analysis showed an excellent agreement, confirmed by very low mean of differences and absence of recognizable trends at visual inspection of scatter plots, with constant variance across the measuring range.

### Evaluation of fluid dynamics in low-flow low-gradient aortic stenosis

Within the AS group, 10 patients were diagnosed with LFLG AS. These had lower baseline mean gradient (26.2 ± 3.8 mmHg, *P* < 0.001) and a lower LVEF (36.9 ± 6.7%, *P* < 0.001) compared with the remaining AS patients with classical high-gradient normal flow (NFHG). Patients with LFLG showed similar changes of intracardiac flow dynamics to those observed with NFHG AS patients. In particular, LFLG presented with significantly higher values of VL (*P* = 0.003) and KED (*P* = 0.001), compared with CTRLs. On the contrary, although VD showed a similar numerical change in the LFLG subgroup, this difference did not reach the threshold for significance compared with CTRLs (*P* = 0.056). Nevertheless, VD (optimal cut-off point = 0.354; sensitivity 71%; specificity 77%) was able to correctly reclassify 70% of the patients that would have been wrongly under-classified using the standard echocardiographic parameters (mean gradient, Vmax, and AVA), being LFLG severe AS patients.

## Discussion

The present study describes, for the first time, the intracardiac flow properties in patients with severe AS when compared with CTRLs and patients with LV concentric remodelling in absence of AS. As LV remodelling inevitably accompanies AS, this latter group was included in the study to understand whether the observed changes in fluid dynamics were mainly related to the valvular stenosis itself or there was a possible impact of LV remodelling, independently of the valvular stenosis.

The main findings of the present study are as follows: (i) the study of intracardiac flow dynamics by means of HyperDoppler is feasible with an excellent reproducibility in patients with severe AS; (ii) a significant change of vortex localization, vorticity, and energy parameters distinguishes patients with AS from CTRLs; (iii) VD can differentiate patients with AS from both CTRLs and patients with concentric LV remodelling without AS, with high accuracy; and (iv) patients with a LFLG AS present with similar changes in fluid dynamics, both in localization and energy parameters, as patients with NFHG.

### The fluid dynamics intracardiac flow properties

The heart is a changeful system where intracardiac flow forces are exchanged continuously between the flowing blood and the surrounding tissues. Hence, the flourishing interest in the contribution of vortices to cardiac function probably raises from their potential to explore cardiac mechanics. During the past 20 years, phase contrast cardiac magnetic resonance or contrast echocardiography with particle imaging velocimetry (echo-PIV) been employed to perform intracardiac flow analysis for the assessment of cardiac function.^13,14^ However, these technologies were not able to enter the clinical routine, being complex, time-consuming, and of limited availability. Furthermore, use of ultrasound contrast for echo-PIV studies is expensive and unfeasible in several settings.

Most recently, the introduction of colour Doppler–based ultrasound techniques, which allow an easy reconstruction of the velocity vector field in a semi-automated and contrast-free way, have renewed enthusiasms and expectations on the possibility of convert intracardiac vortex assessment into routinary quantification of cardiac function.^3,15^

### Vortex flow dynamics in patients with aortic stenosis

HyperDoppler modality allows a quantitative characterization of the morphology, size, position, and energy of the vortex structure within the LV. In a hypertrophied LV with severe AS, we found that the vortex flow profile has greater VA, VL, and VD compared with normal subjects. Vortex intensity is also increased in AS. In summary, vortex dynamics in patients with AS is characterized by a larger vortex that is displaced towards the apex of the LV.

Moreover, the vortex profile in AS differentiates from that of patients with only concentric remodelling in absence of AS, who presented with significantly reduced VD and intensity parameters compared with the former. *Videlicet*, VR patients have a more physiological fluid dynamics profile, with the main vortex less displaced towards the apex.

The geometrical vortex profile we observed in AS patients differentiates also from those described in athletes by Fiorencis *et al*.,^15^ with an increased VA but physiological location within the LV cavity (not displaced towards the apex). On the contrary, our findings in CTRLs are consistent with those reported by Fiorencis *et al*. in sedentary subjects with no cardiac disease.

Our findings are also in agreement with what already observed by Cimino *et al*.^16^ using the echo-PIV technique with contrast administration. The authors demonstrated the existence of a LV intracavitary pressure gradient, mainly located in healthy volunteers at basal level and directed towards the apex with changes in both location and size according to changes in LV geometry.

Our finding of a higher mean KED exclusively in the AS group might reflect the increased turbulence, a common finding in the presence of an increased afterload and a restricted flow as can be observed in case of AS but not in isolated LV concentric remodelling without outflow obstruction.

Prior studies have investigated the role of turbulent KE (TKE) and of the degree of turbulence in the characterization of aortic valve stenosis^17^ and the role of four-dimensional (4D) flow magnetic resonance imaging (MRI)–based turbulence mapping to determine the irreversible energy loss over a stenosis.^18^ A study in 51 patients with AS and 10 CTRLs by Binter *et al*.^19^ has shown that 4D flow-derived TKE is significantly higher in patients with AS when compared with CTRLs and is able to provide complementary information to echocardiography for the determination of AS severity.

Moreover, the KED behaviour by HyperDoppler was previously assessed in cardiomyopathies by Mangual *et al*.^20^ who reported reduced values of KED in patients with dilated cardiomyopathy in comparison with healthy subjects, most probably result of a reduced KE in relation to the reduced force of contraction.

Conversely, Fiorencis *et al*. demonstrated a higher KED by HyperDoppler in athletes, depending on the higher flow velocities of early LV filling and in line with the observations of Steding-Ehrenborg *et al*.^21^ who demonstrated a higher KE at early diastole in athletes compared with CTRL patients, as a result of enhanced diastolic function.

In line with other contemporary studies on fluid dynamics, our data strongly support the concept that KED together with the analysis of intra-ventricular vortex geometries (such as VD) might provide complementary information to the standard echocardiographic analyses on AS, helping to discriminate within the heterogeneous population of patients with AS. For example, KED may fit as surrogate for the haemodynamic severity of AS in patients with discrepant echocardiographic values of mean pressure gradient and AVA, or in those with confounding diseases unambiguously attributed to AS, or in patients who do not qualify for exercise testing to unmask exercise-induced symptoms. Furthermore, additional information from intracardiac fluid dynamics might provide useful data to improve the assessment of patients with AS and diverse degrees of VR such those with cardiac amyloidosis, or Anderson–Fabry,^22–24^ with the potential of improving both the clinical management and the prognosis.^25^

### Fluid dynamics assessment in patients with low-flow low-gradient aortic stenosis

An important number of patients with AS have a ‘low-gradient’ AS, which means a small AVA (<1.0 cm2) coherent with severe AS but a low mean transvalvular gradient (>40 mmHg) coherent with non-severe AS. These include three main subtypes: (i) classical (reduced LVEF) low-flow, low-gradient (LFLG), (ii) normal-flow, low-gradient (NF-LG) AS, and (iii) paradoxical (preserved LVEF) LFLG.

Diagnosis of AS severity in this subset of patients is particularly challenging, as the AVA-gradient discrepancy is often susceptible of technical pitfalls and measurement errors; indeed, low-dose DSE and/or the quantitation of the degree of aortic valve calcification by multi-detector computed tomography is often needed to corroborate AS severity. The uncertainty about the actual stenosis severity, in turn, raises perplexity about the indication for aortic valve replacement when the patient has symptoms and/or LV systolic dysfunction.

In our study, the subset of patients with LFLG AS exhibits the same pattern with no significant variation of both morphology and energy parameters of fluid dynamics compared with NFHG AS, with a lower DF compared with the latter, even whether not statistically significant. In particular, as for NFHG, LFLG manifested a statistically significant increase of VL and energy dissipation (*P* = 0.003 and *P* = 0.001, respectively) compared with CTRL. In addition, the finding that VD was able to correctly reclassify 70% of the LFLG severe AS patients, therefore adding a relevant piece of information to the standard diagnostic parameters merits further investigation in a larger population to specifically focus on the incremental value of cardiac fluid dynamics over known metrics for assessing AS severity and predicting the development of clinical end points in this particular subset of AS patients.

The evolution and diffusion of such novel fluid dynamics parameters may be momentous for optimizing the timing of therapeutic interventions and architecting repair strategies to avoid irreversible changes in cardiac structure and function.

### Study limitations

This is a single-centre study. However, despite more studies are certainly needed to establish the exact role of quantitative vortex analysis in this cohort of patients, changes in fluid dynamics were consistently observed in our relatively small cohort. Aortic stenosis is often coexistent with other significant valvular heart disease, and this may impact on intracardiac fluid dynamics analysis. In our study, we tried to account for this potential confounding effect, excluding patients with concomitant significant valvopathies. The fluid dynamics readings obtained by means of the HyperDoppler software provide a global measure of the vortex pertaining to the entire heartbeat. On one hand, this represents a strength, since it reduces the variability of the measurements, providing more reliable readings. In fact, measuring vortex properties frame by frame can be subjected to a large variability and consequently difficult reproducibility. However, on the other hand, it is possible that additional details might be obtained by measuring flow dynamics frame by frame. However, this can be subjected to a large variability and difficult reproducibility with currently available methodologies. Finally, CDFM technique has not been validated against phase contrast magnetic resonance imaging (PC-MRI). However, its accuracy compared with echo-PIV technique has been previously demonstrated, using a transparent LV phantom, allowing simultaneous acquisition and comparison of both colour flow mapping and echo-PIV data.^26^ Of note, the latter has been also validated against PC-MRI.^27^

## Conclusions

To the best of our knowledge, this is the first study providing a non-invasive quantitative assessment of intracardiac fluid dynamics in patients with severe AS using CDFM. There is a significant change of vortex localization, vorticity, and energy parameters in patients with AS. In particular, VD, VI, and energy dissipation were all significantly increased in AS compared with CTRLs. Vortex depth was also able to discriminate with accuracy patients with AS. These results may warrant further studies in order to establish the incremental value of this additional analysis to the baseline echocardiographic assessment.

## Clinical perspectives

This work highlights the potential role of a novel echocardiographic technique allowing non-invasive and quantitative fluid dynamics analysis in patients with severe AS. Further studies are needed to establish the prognostic role of this non-invasive indices and their correlation with other cardiac biomarkers. Despite promising, these quantitative parameters should be also compared with the current gold standard for fluid dynamics analysis: cardiac magnetic resonance.

## Supplementary Material

oeae069_Supplementary_Data

## Data Availability

The data underlying this article will be shared on reasonable request to the corresponding author.

## Lead author biography

Dr Jolanda Sabatino is a cardiovascular physician and researcher, renowned for groundbreaking contributions to paediatric cardiology and advanced echocardiography techniques. Graduating magna cum laude from Magna Graecia University of Catanzaro, she continued her academic journey with a PhD in biomarkers of chronic diseases, focusing on identifying LV non-compaction in childhood. With extensive clinical experience at prestigious institutions like the Royal Brompton Hospital in London and the University of Padua in Italy, Dr Sabatino at her young age has excelled as a researcher, clinician, and educator. Her prolific research output has earned numerous awards, including grants from esteemed organizations like the European Society of Cardiology and the Italian Society of Cardiology.
