# Supplementary material for: Changes of intracardiac flow dynamics measured by HyperDoppler in patients with aortic stenosis
Source: Eur Heart J Open. 2024 Aug 8;4(5):oeae069. doi: 10.1093/ehjopen/oeae069 (PMC11369357; doi:10.1093/ehjopen/oeae069)
Supplement: oeae069_Supplementary_Data [file oeae069_supplementary_data.docx]

**Supplementary Table 1. Reproducibility of Fluid dynamics parameters**

| **Vortex parameters** | **ICC (95% CI)** |
| --- | --- |
| **Vortex_Area** | 0.909 (0.863-0.940) |
| **Vortex_Intensity** | 0.816 (0.722-0.878) |
| **Vortex_Depth** | 0.949 (0.923-0.966) |
| **Vortex_Lenght** | 0.915 (0.871-0.944) |
| **Energy_Dissipation** | 0.919 (0.878-0.946) |
| **Vorticity_Fluctation** | 0.896 (0.843-0.931) |
| **Kinetic_Energy_Fluctation** | 0.909 (0.862-0.939) |
| **Shear_Stress_Fluctation** | 0.868 (0.802-0.913) |
| **Flow_Force_Parameter** | 0.813 (0.717-0.876) |
| **Flow_Force_Angle** | 0.811 (0.715-0.875) |
| **Direct_Flow** | 0.393 (0.076-0.610) |
